# Dynamics of mtDNA introgression during species range expansion: insights from an experimental longitudinal study

**DOI:** 10.1038/srep30355

**Published:** 2016-07-27

**Authors:** V. Mastrantonio, D. Porretta, S. Urbanelli, G. Crasta, G. Nascetti

**Affiliations:** 1 Department of Environmental Biology, Sapienza University of Rome, Rome, Italy; 2 Department of Mathematics, Sapienza University of Rome, Rome, Italy; 3 Department of Ecological and Biological Sciences, Tuscia University, Viterbo, Italy

## Abstract

Introgressive hybridization represents one of the long-lasting debated genetic consequences of species range expansion. Mitochondrial DNA has been shown to heavily introgress between interbreeding animal species that meet in new sympatric areas and, often, asymmetric introgression from local to the colonizing populations has been observed. Disentangling among the evolutionary and ecological processes that might shape this pattern remains difficult, because they continuously act across time and space. In this context, long-term studies can be of paramount importance. Here, we investigated the dynamics of mitochondrial introgression between two mosquito species (*Aedes mariae* and *Ae. zammitii *) during a colonization event that started in 1986 after a translocation experiment. By analyzing 1,659 individuals across 25 years, we showed that introgression occurred earlier and at a higher frequency in the introduced than in the local species, showing a pattern of asymmetric introgression. Throughout time, introgression increased slowly in the local species, becoming reciprocal at most sites. The rare opportunity to investigate the pattern of introgression across time during a range expansion along with the characteristics of our study-system allowed us to support a role of demographic dynamics in determining the observed introgression pattern.

Geographic range is naturally dynamic, and it has constantly changed in the history of several species. In the past, climatic oscillations led species to periodically contract and re-expand their geographic ranges in response to the environmental conditions^1^. Currently, natural and human-mediated increases in global temperatures, along with anthropogenic disturbances, are rapidly promoting range expansion, via the change or breakdown of habitat barriers^2^,^3^. The consequences of an increased connection between populations remains a challenging question for evolutionary and ecological sciences^4^.

When previously allopatric and not fully reproductively isolated populations meet in new overlapping areas, they can hybridize and genes can cross species boundaries^5^. Introgressive hybridization, described as the permanent incorporation of genes from one set of differentiated populations into another^6^, represents a long-lasting debated consequence of species range expansion. Although considered for a long time as a minor process in nature, currently, it is well-recognized that introgression is widespread. Divergent populations, subspecies, and closely and ancient related species have been shown to hybridize and introgress after range expansion, in both plant and animal kingdoms^7^.

In the last few decades, main interest about introgression was focused to understand its genetic pattern. Empirical studies, particularly involving hybrid zones of secondary contact, have shown that introgression rate and its geographic extent can be highly variable among genomes (i.e. organelle *vs.* nuclear genome) as well as among different regions of the same genome^8^,^9^. In animals, mitochondrial DNA (mtDNA) is more prone to introgress than the nuclear genome, to such an extent that complete mtDNA replacement has been observed even in the presence of little or no nuclear introgression^10^,^11^,^12^. Furthermore, mtDNA introgression was often asymmetric between populations (i.e. from one lineage to another) and mostly occurring from local to colonizing species [see 10 for a review^13^,^14^,^15^].

Deterministic and stochastic processes have been inferred to explain asymmetric introgression of mtDNA. Natural selection has often been invoked as the driver of the increased frequency of adaptive introgressed mitochondrial variants^16^,^17^,^18^. In several other cases, since mtDNA is maternally inherited, differences in population size or differential production of offspring have also been invoked^19^. More recently, a major role of demographic dynamics occurring during range expansion has been suggested to explain mtDNA introgression from local to colonizing species^20^,^21^,^22^, but see ref. 23.

Some difficulties in understanding the above mentioned processes are due to the fact that most of the previous studies, which focused on single time points, offered single and, often, *a posteriori* snapshots of this process, whereas introgression develops in a dynamic and complex spatio-temporal context where several factors can operate continuously and at different stages, influencing the final genetic pattern of introgression^8^,^24^,^25^. Furthermore, complementary data that could help in understanding the different processes (i.e. dispersal, mating behavior, or offspring fitness) is often lacking for many natural study systems^10^.

Recently established hybrid zones (e.g. as a consequence of human-mediated introduction) are potentially good study systems for investigating the spatio-temporal dynamics of introgression since the early stages of contact^8^. In this context, the artificial sympatric area between the mosquitoes *Aedes mariae* and *Ae. zammitii* offers a unique opportunity. They are two sibling species that develop in the water of rock pools along the Mediterranean Basin^26^, and that have a strict coastal distribution: *Ae. mariae* is distributed along the western Mediterranean coasts, while *Ae. zammitii* inhabits the central and eastern coasts^26^,^27^,^28^,^29^. In June 1986, an artificial sympatric area between the two species was originated along the Adriatic Sea coasts by translocating individuals of *Ae. mariae*, collected at two localities along the Tyrrhenian Sea, into the locality Baia dei Campi, within the geographic range of *Ae. zammitii* (Fig. 1)^29^. Several features make this study system suitable for analyzing mtDNA introgression across time during a range expansion. First, after its introduction, *Ae. mariae* diffused from the site of initial release and colonized the neighboring sites; in 2011, the species was present along a transect of about 20 km, coexisting in syntopy with *Ae. zammitii.* Second, reproductive isolation between *Ae. mariae* and *Ae. zammitii* is not complete, and persistent hybridization has been observed in the sympatric area across the years^29^. Although F1 hybrid males are sterile, F1 hybrid females (from both ♂ *mariae* × ♀ *zammitii* and ♂ *zammitii* × ♀ *mariae* crosses) are vigorous, fertile, and able to backcross with both parental species, allowing gene introgression between the two species. Our preliminary data using nuclear allozyme markers showed the occurrence of admixed individuals in both species, which supports that backcrosses and introgression actually occur in nature^26^,^28^,^29^,^30^. Third, individuals of *Ae. mariae* and *Ae. zammitii* were collected across the sympatric area from their first contact until recently (from 1986 to 2011), covering approximately 25 years and 210 generations^29^. This sampling scheme allowed us to investigate not only the current pattern of mtDNA introgression but also its temporal dynamics during the colonization process.

In this paper we aimed to test: (*i*) if mtDNA introgression occurs between *Ae. mariae* and *Ae. zammitii*; (*ii*) if introgression is asymmetric between them; and (*iii*) if so, if it was biased from the local (*Ae. zammitii*) to the colonizing species (*Ae. mariae*). Finally, the possible factors underlying the observed genetic pattern were discussed.

## Results

**Figure 1:**
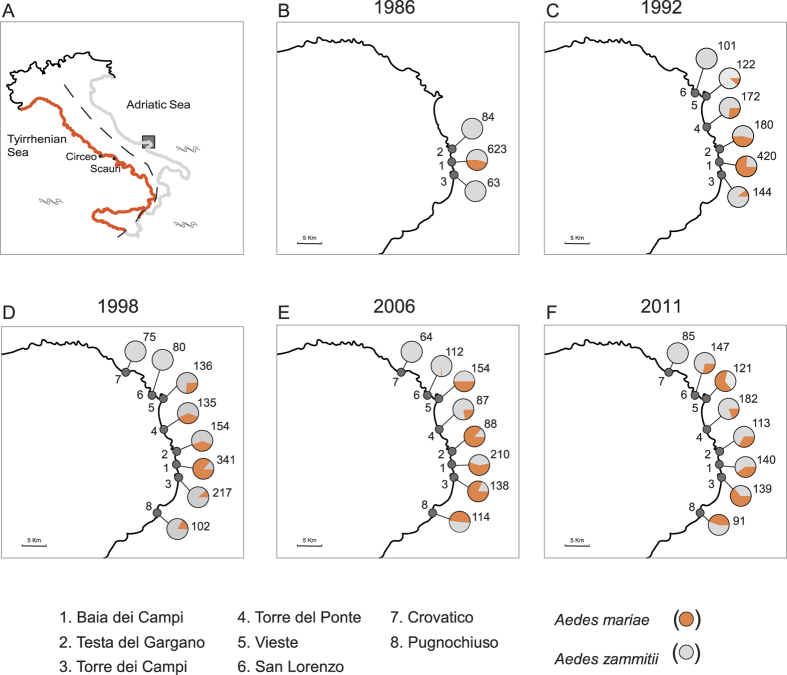
Sampling localities of the sympatric area. Panel (**A**): geographic distribution of *Aedes mariae* (orange) and *Ae. zammitii* (light grey) along the coasts of the Italian Peninsula. The populations of *Ae. mariae* (Circeo and Scauri) used in the translocation experiment are also shown. Panels (**B–F**): sampling localities, years of collection and proportions of *Ae. mariae* (orange) and *Ae. zammitii* (light grey) in each locality. The number of *Ae. mariae* and *Ae. zammitii* individuals found are also shown (data from ref. 29). The sketch map was drawn using the software Canvas 15 (ACD systems http://www.acdsee.com/de/products/canvas-15) by tracing a map of the Gargano Promontory generated in QGIS 2.12 (http://www.qgis.org/it/site).

Since the introduction of *Aedes mariae* in June 1986, the proportion of *Ae. mariae* and *Ae. zammitii* changed across time (Supplementary Table S1). Indeed *Ae. mariae* spread across the coastline and increased its frequency in the newly colonized localities (Fig. 1). To assess mitochondrial introgression, we analyzed a total of 1,659 individuals (976 *Ae. zammitii* and 683 *Ae. mariae*) collected from the sympatric area from October 1986 to 2011 (Supplementary Table S2). These individuals were genotyped at allozymic loci and identified as *Ae. mariae* or *Ae. zammitii* in our previous study^29^. Here, we considered as *introgressed* the individuals genotyped as *Ae. mariae* and *Ae. zammitii* at allozymes and harboring haplotypes characteristic of the other species at mtDNA. Of the 976 *Ae. zammitii* individuals analyzed, 32 were introgressed. Of the 683 *Ae. mariae* individuals analyzed, 98 were introgressed.

A subset of pure *Ae. mariae* and *Ae. zammitii* individuals (25 individual for each species) and all mtDNA introgressed individuals were sequenced to check for consistency with the restriction pattern observed. In all cases, we found congruence between the RFLP patterns and sequencing results. In the 25 pure *Ae. mariae* individuals sequenced, four haplotypes (m3, m4, m6 and m8) were found that are characteristic of the Circeo and Scauri allopatric populations (i.e. the populations used for the translocation experiment)^26^. Likewise, in the 25 pure *Ae. zammitti* individuals sequenced, four haplotypes (z1, z2, z3 and z5) were observed that were found also in the Peschici and Baia dei Campi populations^26^ (Supplementary Table S3 and Figure S1). In the introgressed individuals of both *Ae. mariae* and *Ae. zammitii* different haplotypes of the other species were found (Supplementary Table S2). In particular, in the *Ae. mariae* introgressed individuals the haplotypes z1, z2, z3, z4 and z5 were found that are characteristic of the Peschici and Baia dei Campi populations^26^.

**Figure 2:**
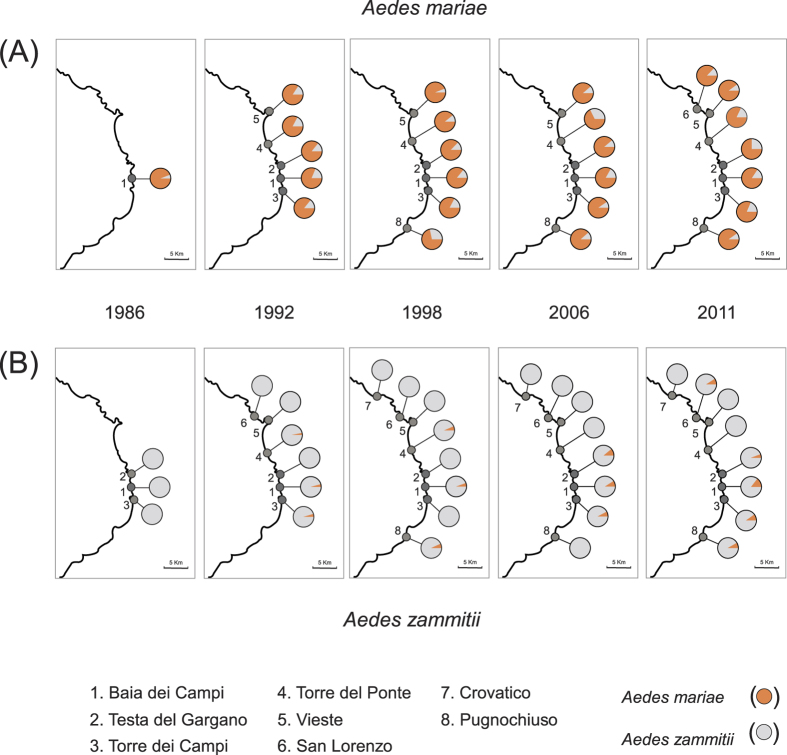
Proportion of mitochondrial DNA haplotypes found in the sympatric area. Panel (**A**) *Aedes mariae*, Panel (**B**) *Ae. zammitii.* The characteristic haplotypes of *Ae. mariae* and *Ae. zammitii* are shown in orange and light grey, respectively. The sketch map was drawn using the software Canvas 15 (ACD systems http://www.acdsee.com/de/products/canvas-15) by tracing a map of the Gargano Promontory generated in QGIS 2.12 (http://www.qgis.org/it/site).

**Figure 3:**
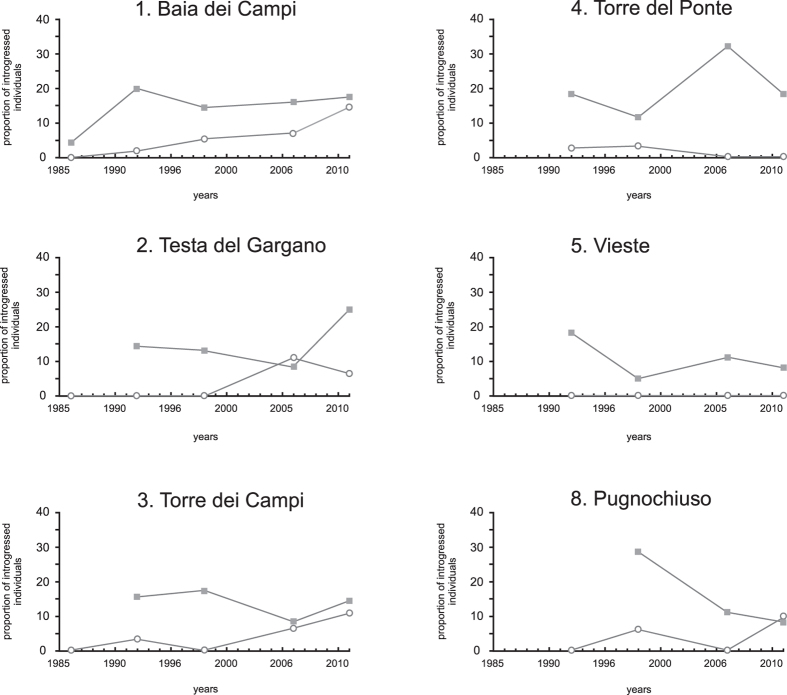
Proportion of the mtDNA introgressed individuals of *Aedes mariae* (square) and *Ae. zammitii* (circle) found in each site of the sympatric area. Fit with constant distribution was found using the goodness-of-fit test for introgressed individuals of *Aedes mariae* (square) in the site1 Baia dei Campi (data from 1986 were excluded) (*χ*^2^ = 0.40 *P* = 0.820).

Introgression of *Ae. zammitii* mtDNA into *Ae. mariae* was detected as early as 1986 at the release site Baia dei Campi (4.4%) (Fig. 2A). In 1992, the proportion of mtDNA introgressed individuals of *Ae. mariae* ranged from 11.4% (site 2, Testa del Gargano) to 20% (site 1, Baia dei Campi); in 1998, from 5% (site 5, Vieste) to 28.6% (site 8, Pugnochiuso); in 2006, from 8% (site 3, Torre dei Campi) to 30% (site 4, Torre del Ponte); and in 2011, from 6.5% (site 5, Vieste) to 18.4% (site 4, Torre del Ponte). The analysis of the introgression across time showed no time dependence at any site (Fig. 3 and Supplementary Table S4).

Introgression of *Ae. mariae* mtDNA into *Ae. zammitii* was detected as early as 1992 (Fig. 2B). In 1992, mtDNA introgressed individuals of *Ae. zammitii* were found at three sites of the sympatric area (site 1, Baia dei Campi; site 4, Torre del Ponte; and site 3, Torre dei Campi), and their number ranged from 2.5% to 3.4%. In 1998, introgression was found at three sites with values ranging from 3.1% (site 4, Torre del Ponte) to 6.2% (site 8, Pugnochiuso); in 2006, it was 7.4% (site 1, Baia dei Campi), 11% (site 2, Testa del Gargano), and 6.6% (site 3, Torre dei Campi); finally, in 2011, introgression ranged from 5.7% (site 2, Testa del Gargano) to 14.3% (site 1, Baia dei Campi). No time dependence was found at any site, with the exception of site 1, Baia dei Campi (*P* < 0.05; Supplementary Table S4 and Fig. 3). Logistic regression performed to compare the proportion of mtDNA introgression into *Ae. mariae* vs *Ae. zammitii* showed a dependence with respect to species in all sites and with respect to years in the site Baia dei Campi (Supplementary Table S5).

## Discussion

Translocation and expansion of *Aedes mariae* into the range of *Ae. zammitii* allowed two allopatric interbreeding species to meet in the same area^29^. This artificial sympatric area, because of its experimental nature, has offered the rare opportunity to investigate the genetic consequences of range expansion since the early stages of this process.

Our genetic survey of sympatric populations showed the sharing of mtDNA haplotypes between *Ae. mariae* and *Ae. zammitii* (Fig. 2). We can attribute this sharing to gene introgression because of the following reasons: (*i*) reproductive isolation between the two species is not complete, and hybridization occurs in the sympatric area^29^; (*ii*) F1 hybrid males are sterile, but F1 hybrid females from both crosses (i.e., ♂ *mariae* × ♀ *zammitii* and ♂ *zammitii* × ♀ *mariae*) are vigorous, fertile, and able to backcross with both parental species, allowing mtDNA introgression^27^,^29^; (*iii*) no mtDNA haplotypes of *Ae. mariae* were found in *Ae. zammitii* populations either in the release site Baia dei Campi before the translocation experiment in 1986 and in any other allopatric population studied; no mtDNA haplotypes of *Ae. zammitii* were found in *Ae. mariae* populations used for translocation (Circeo and Scauri) and in any other allopatric population studied^26^; *iv*) the occurrence of incomplete lineage sorting can be excluded as the analysis of mtDNA across the geographic ranges of the two species showed that they were reciprocally monophyletic and diverged during the early Pleistocene^26^.

The analysis of the introgression pattern at mtDNA between *Ae. mariae* and *Ae. zammitii* showed significantly different proportions of mtDNA introgression between the two species (Supplementary Table S5). At all sites analyzed and across the whole sympatric area, introgression was found earlier and at higher frequency into the introduced species *Ae. mariae* than in the local species *Ae. zammitii* (Figs 2 and 3; Supplementary Tables S2 and S4). However, with the passage of generations, the proportion of introgressed individuals into *Ae. zammitii* increased slowly, reaching, in some localities, similar values to those found in *Ae. mariae* (Figs 2 and 3).

Among the factors inferred to explain asymmetric introgression of mtDNA, demographic dynamics involving the species during a range expansion have been stressed upon. Using spatially explicit simulations of haploid genomes, Currat *et al*.^20^ modeled a species that invades an occupied area and interacts with local populations by interbreeding with them. According to their results, under neutral conditions, asymmetric mtDNA introgression may occur from the local to the colonizing species and is a consequence of spatial dynamics underlying the colonization process^20^,^31^. During a range expansion, differential demography characterizes the local and colonizing species in the expansion wave. Indeed, contrary to local populations that are already at carrying capacity, colonizing populations are still at very low density and demographically grow. As a consequence, the frequency of introgressed genes in the colonizing species is amplified in the initial phases of the expansion, leading to asymmetric introgression from the local to the colonizing species^20^.

The introgression pattern and its dynamics across time observed between *Ae. mariae* and *Ae. zammitii* seem to fit well with the above mentioned expectations (Fig. 2). Since its introduction in 1986, *Ae. mariae* spread across the coastline and increased its frequency in the newly colonized localities (Fig. 1, Supplementary Table S1). The growth of populations at the wave front of colonization could therefore account for the observed phase of asymmetric introgression from local species. Likewise, demographic dynamics could account for the genetic pattern observed in *Ae. zammitii*. In contrast to what happened in *Ae. mariae*, introgression into the local species did not spread rapidly because local populations were demographically stable and the introgressed mtDNA haplotypes were likely diluted by gene flow from neighbouring sites. However, diffusion of the colonizing species allowed recurrent interbreeding events, which would progressively increase the extent of introgression in the local species.

Factors other than demographic dynamics, however, could explain mitochondrial asymmetric introgression, as for example, differences in population size^10^. In our system, both pre- and post-mating isolation barriers occur between the two species, which partially prevent hybridization (i.e. different height of mating swarms and sterility of F1 hybrid males). However, in conditions of high demographic disparity, the extent of discrimination can be relaxed in females of rare species, as a consequence of poor availability of conspecific males, whereas, it does not change for the more abundant species, as conspecific males are present^32^. Thus, introgression from the colonizing species *Ae. mariae* to the local species *Ae. zammitii* would be expected in our system. Actually, the observed genetic pattern showed the opposite direction of introgression, namely, from the more abundant species *Ae. zammitii* to the rare species *Ae. mariae*. Therefore, differences in population size seems quite unlikely to explain the observed asymmetric mtDNA introgression. Differential production of offspring is an alternative factor commonly invoked to explain asymmetric introgression of mtDNA. According to this hypothesis, if a detrimental heterospecific combination occurs after hybridization, few or no offspring can result from the crosses^10^,^32^. In these conditions, backcrosses accumulate in only one direction, leading to asymmetric mtDNA introgression. Our genetic survey of the sympatric area showed, in both species, the occurrence of F1 hybrids, backcrossed and parental individuals harboring mtDNA of both *Ae. mariae* and *Ae. zammitii*, indicating that heterospecific crosses can occur in both directions (i.e. ♀ *Ae. mariae* × ♂ *Ae. zammitii* and ♀ *Ae. zammitii* × ♂ *Ae. mariae*) as well as backcrosses can occur with both parental species. Furthermore, we found that throughout time mtDNA introgression into *Ae. zammitii*, in some localities, was similar to that found into *Ae. mariae*, which makes this hypothesis quite unlikely.

Finally, asymmetric mtDNA introgression can be attributed to adaptive processes^10^. In ephemeral pools, several intra- and inter-specific interactions may occur between all life stages of mosquitoes. In adult females, predation and cannibalism of older larval stages on eggs or younger larvae have been shown to affect oviposition habitat selection^33^,^34^,^35^,^36^. Likewise, in larval stages, competition for food and space and predation, scavenger, and cannibalistic behaviors have been shown to frequently occur and affect not only individual fitness but also population dynamics and species abundance^37^. The mitochondrial genome plays a central role in the production of cellular energy and can affect several life-history traits, encompassing lifespan, fertility, and starvation resistance^38^,^39^,^40^. Because some components of oxidative phosphorylation as well as some factors involved in mtDNA transcription and translation are encoded by the nuclear genome, mtDNA introgression may affect mitonuclear interactions, leading to differences in fitness-related life-history traits among parental and introgressed individuals^41^,^42^,^43^,^44^,^45^. More recently it has been also hypothesized that spatial sorting of behavioural polymorphisms during range expansion could affect the mtDNA introgression pattern during the subsequent sympatric phases^46^ as well as that the sorting of dispersal related traits could affect the spread of introgressed individuals^47^. At present, no studies have been focused on the relative dispersal or competitive abilities of *Ae. mariae* and *Ae. zammitii*. At this stage of investigations, further studies about relative fitness between parentals and mtDNA introgressed individuals of *Ae. mariae* and *Ae. zammitii* are required to investigate the possible contribution of selective factors along with demographic processes. In this context, the analysis of introgression at nuclear genome by next-generation sequencing technologies could be particularly useful in investigating how introgression affects neutral and functional regions within the nuclear genome as well as the possible occurrence of mitonuclear co-adaptation.

## Conclusions

The *Ae. mariae/Ae. zammitii* case study is among the few cases in which the involvement of demographic processes in shaping the observed pattern of asymmetric introgression is more than just a speculative or supplementary hypothesis proposed along with deterministic factors, as is often observed in single time point or *a posteriori* studies^10^,^20^,^31^. Indeed, analysis across time during species expansion, reciprocal introgression observed with the passage of generations, and the peculiar features of the study system allowed us to discard some hypotheses and support the role of demographic dynamics in determining the observed pattern of mtDNA asymmetric introgression.

## Materials and Methods

### Sampling

*Aedes mariae* and *Ae. zammitii* are morphologically indistinguishable species, but they are well characterized by both nuclear and mitochondrial genetic markers. Six discriminative allozymic loci (i.e. with fixed alternative alleles in the two species) and two distinct pools of mitochondrial DNA haplotypes have been found in populations of the two species across their geographic range^26^,^29^.

In June 1986, an artificial sympatric area was created between these two species by translocating individuals of *Ae. mariae* into the geographic range of *Ae. zammitii*. In our previous study, we analysed at allozymic loci about 16,000 individuals sampled in the sympatric area from October 1986 (i.e. about eight generations after release) to 2011^26^,^29^. Here, we used a random sample of the individuals collected in 1986, 1992, 1998, 2006, and 2011, that were genotyped at allozymic loci and recognized as “pure” *Ae. mariae* or *Ae. zammitii* (i.e. individuals that have only the alleles characteristic of *Ae. mariae* or *Ae. zammitii* at all the six discriminative allozymic loci, as described in Urbanelli *et al*.^29^). A total of 1,659 individuals were analysed to assess mtDNA introgression.

### Laboratory procedures and data analysis

The analysis of mtDNA across the geographic ranges of the species revealed the occurrence of two distinct pools of haplotypes between *Ae. mariae* and *Ae. zammitii*^26^. On the basis of the COI sequences (GenBank accession number KM592029–KM592055), using the software DNA for Windows (www.dna-software.co.uk), we found that the *BspLi*/*Nla*IV restriction enzyme (5′-GGN|NCC-3′) clearly discriminated between the mtDNA haplotypes of the two species (Supplementary Figure S1). Therefore, we used PCR-restriction fragment length polymorphisms (RFLPs) to identify mtDNA of *Ae. mariae* and *Ae. zammitii* individuals sampled in the sympatric area.

Total genomic DNA was extracted from the mosquito abdomen, which was excised before allozymic analyses, and stored in single eppendorf tubes at −80 °C^29^. The standard cetyltrimethyl ammonium bromide (CTAB) protocol was followed to extract DNA^48^.

PCR amplification of the COI mitochondrial gene fragment was performed using the primers pairs *mzp-*COI-f 5′-TTTTCGGAGTTTGATCAGGAA-3′ and *mzp-*COI-r5′-TTCAGGATGTCCAAAGAATCAA-3′^49^. PCR cycling procedure was: 95 °C for 5 min followed by 34 cycles of 93 °C for 1 min, 55 °C for 1 min, 72 °C for 1 min 30 s, and then by a single, final step at 72 °C for 10 min. PCR products were digested with *BspLi*/*Nla*IV (Dasit, Milan, Italy), according to the manufacturer’s protocol: the digestion reaction mixture was prepared using 12 μL PCR product, 0.1 μL *BspLi*/*Nla*IV (10 U/μL), dH_2_O and Tango buffer, and was incubated at 37 °C overnight. Digestion products were separated by electrophoresis on a 2.0% agarose, 0.5X TAE gel, and visualized by staining with Gelred (Sigma-Aldrich, Milan, Italy). The sizes of the DNA fragments were assessed using the 100 bp DNA ladder (Promega, Milan, Italy) run on the same gel.

Twenty-five *Ae. mariae* individuals and 25 *Ae. zammitii* individuals collected at Baia dei Campi (5 individuals of each species for each year), and all mtDNA introgressed individuals were sequenced to check for consistency with the restriction pattern observed. PCR sequences were obtained using the ABI PRISM 3700 DNA sequencer by Macrogen Inc. (www.macrogen.com) and edited using the software chromas 2.31. Then, the sequences were aligned to the sequences of COI fragments deposited in GenBank by using clustal × 2.0. We considered the individuals of *Ae. mariae* and *Ae. zammitii* that had mtDNA haplotypes characteristic of the other species as *introgressed* (see also the Discussion section).

Logistic regression was performed *i*) to assess changes of the proportions of *Ae. mariae* vs *Ae. zammitii* across years in each site; *ii*) to assess changes of the proportion of introgressed individuals within each species at each site and for each year; *iii*) to compare the proportions of mtDNA introgression into *Ae. mariae* vs *Ae. zammitii*. All analyses were performed using the software R 2.6.2^50^.

## Additional Information

**How to cite this article**: Mastrantonio, V. *et al*. Dynamics of mtDNA introgression during species range expansion: insights from an experimental longitudinal study. *Sci. Rep.* **6**, 30355; doi: 10.1038/srep30355 (2016).

## Supplementary Material

Supplementary Information
