# Supplementary material for: Dynamics of mtDNA introgression during species range expansion: insights from an experimental longitudinal study
Source: Sci Rep. 2016 Jul 27;6:30355. doi: 10.1038/srep30355 (PMC4962091; doi:10.1038/srep30355)
Supplement: Supplementary Information [file srep30355-s1.doc]

**Dynamics of mtDNA introgression during species range expansion:**

**insights from an experimental longitudinal study**

Mastrantonio V^1*^, Porretta D ^1*^, Urbanelli S ^1‡^, Crasta G ^2^ and G Nascetti^3^

^1^Department of Environmental Biology, Sapienza University of Rome, Rome, Italy

^2^ Department of Mathematics, Sapienza University of Rome, Rome, Italy

^3^Department of Ecological and Biological Sciences, Tuscia University, Viterbo, Italy

**Table S1.** Analysis of the proportion of the number of individuals of *Ae. mariae* across time by logistic regression. *P* < 0.05 denotes dependence with respect to the parameter “year”. The sites are coded as in Fig. 1.

| **Site** |  | **Estim.** | **Std. Err.** | **z value** | **P(>|z|)** |
| --- | --- | --- | --- | --- | --- |
| 6. San Lorenzo | interc. | 1223.46 | 292.44 | 4.184 | 2.87e-05*** |
|  | year | -0.608 | 0.145 | -4.180 | 2.92e-05*** |
| 5. Vieste | interc. | 324.81 | 31.66 | 10.26 | <2 e^-16^*** |
|  | year | -0.162 | 0.016 | -10.25 | <2 e^-16^*** |
| 4. Torre del Ponte | interc. | -55.667 | 23.851 | -2.33 | 0.0196* |
|  | year | 0.028 | 0.012 | 2.372 | 0.0177* |
| 2. Testa del Gargano | interc. | 79.356 | 19.723 | 4.023 | 5.73 e^-05^*** |
|  | year | -0.040 | 0.010 | -4.026 | 5.66 e^-05^*** |
| 1. Baia dei Campi | interc. | -73.886 | 12.972 | -5.696 | 1.23 e^-08^*** |
|  | year | 0.037 | 0.007 | 5.742 | 9.35 e^-09^*** |
| 3. Torre del Ponte | interc. | 421.13 | 31.137 | 13.53 | <2 e^-16^*** |
|  | year | -0.210 | 0.0156 | -13.52 | <2 e^-16^*** |
| 8. Pugnochiuso | interc. | 183.70 | 47.930 | 3.833 | 0.000127*** |
|  | year | -0.0914 | 0.024 | -3.823 | 0.000132*** |

**Table S2.** Individuals of *Aedes mariae* and *Ae. zammitii* analysed in the sites of the sympatric area. The number of introgressed individuals of the two species are shown in square brackets as well as which haplotype were found. The sites are numbered as in Figure 1. The haplotypes were named as in the Fig. S1. m: haplotypes of *Ae. mariae*; z: haplotypes of *Ae. zammitii*.

|  | **1986** | | **1992** | | **1998** | | **2006** | | **2011** | |
| --- | --- | --- | --- | --- | --- | --- | --- | --- | --- | --- |
|  | *Ae.*  *mariae* | *Ae.*  *zammitii* | *Ae.*  *mariae* | *Ae.*  *zammitii* | *Ae.*  *mariae* | *Ae.*  *zammitii* | *Ae.*  *mariae* | *Ae.*  *zammitii* | *Ae.*  *mariae* | *Ae.*  *zammitii* |
| 7. Crovatico |  |  |  |  | - | 20 [0] | - | 22 [0] | - | 19 (0) |
| 6. San Lorenzo |  |  | - | 18 [0] | - | 22 [0] | - | 33 [0] | 24  [3= z1(2), z4] | 52  [5=m4(2), m8(3)] |
| 5. Vieste |  |  | 16  [3= z3(3)] | 35 [0] | 20  [1= z5] | 33 [0] | 27  [3= z1(3)] | 39 [0] | 31  [2= z1, z5] | 28 (0) |
| 4. Torre del Ponte |  |  | 32  [6=z1(3), z2(2), z5] | 40  [1=m8] | 35  [4= z1(3), z2] | 32  [1=m8] | 20  [6= z1(3), z5(3)] | 29 [0] | 38  [7= z1(5), z2(2)] | 27 (0) |
| 2. Testa del Gargano | - | 20 [0] | 35  [4=z2(3), z5] | 30 [0] | 23  [3= z2(3)] | 28 [0] | 9  [1= z3] | 45  [5=m8(4), m4] | 20  [5= z1(2), z2(2), z5] | 35  [2=m3, m8] |
| 1. Baia dei Campi | 45  [2=z1(2)] | 32 [0] | 50  [10=z1(4), z2(5), z5] | 34  [1=m4] | 35  [5=z1(2), z2(3)] | 35  [2= m4, m8] | 24  [4= z2(2), z3, z5] | 27  [2=m3, m8] | 23  [4= z1, z2(2), z5] | 28  [4= m4(2), m8(2)] |
| 3. Torre dei Campi | - | 20 [0] | 20  [3= z1(2), z3] | 29  [1=m8] | 23  [4= z2(3), z3] | 28 [0] | 25  [2= z2, z5] | 30  [2=m8(2)] | 34  [5= z1, z2(3), z3] | 18  [2=m4, m8] |
| 8. Pugnochiuso |  |  | - | - | 21  [6= z1(4), z2, z3] | 32  [2= m8(2)] | 18  [2= z1(2)] | 36 [0] | 35  [3= z1(3)] | 20  [2=m8(2)] |

**Table S3.** Mitochondrial CO I haplotypes found in pure individuals of *Aedes mariae* and *Ae. zammitii*. Five individuals of each species collected in the release site Baia dei Campi (Fig. 1) were sequenced for each year. The haplotypes were named as in the Fig. S1. In brackets, how many times each haplotype was found.

| **Species** | **1986** | **1992** |  | **1998** |  | **2006** |  | **2011** |
| --- | --- | --- | --- | --- | --- | --- | --- | --- |
| *Ae. mariae* | m8(5) | m4, m8(4) |  | m3, m4, m8(3) |  | m4, m8(4) |  | m4(3), m6, m8 |
| *Ae. zammitii* | z1(2), z2(3) | z1, z2(3), z5 |  | z1(4), z2 |  | z1(2), z2, z3, z5 |  | z1, z2(3), z5 |

**Table S4.** Analysis of the proportion of introgressed individuals of *Aedes mariae* and *Ae. zammitii* across time by logistic regression. *P* < 0.05 denotes dependence with respect to the parameter “year”. The sites are coded as in Fig. 1. The analyses were performed for all sites with the exception of Crovatico where no introgression was observed, and San Lorenzo, where introgression was observed only in 2011 (Table S2).

| **Site** |  | **mtDNA introgression**  **from *Ae. zammitii* into *Ae. mariae*** |  | **mtDNA introgression**  **from *Ae. mariae* into *Ae. zammitii*** |
| --- | --- | --- | --- | --- |
|  |  |  |  |  |
| 5. Vieste |  | | interc. | 69.18 | 93.46 | 0.740 | 0.459 | | --- | --- | --- | --- | --- | | year | -0.036 | 0.047 | -0.764 | 0.445 | |  | | interc. | -38.3 | 1.1e7 | 0 | 1 | | --- | --- | --- | --- | --- | | year | 5.5e-3 | 5.8e3 | 0 | 1 | |
| 4. Torre del Ponte |  | | interc. | -40.6 | 58.56 | -0.694 | 0.488 | | --- | --- | --- | --- | --- | | year | 0.019 | 0.029 | 0.668 | 0.504 | |  | | interc. | 346.1 | 368.5 | 0.939 | 0.348 | | --- | --- | --- | --- | --- | | year | -0.175 | 0.185 | -0.949 | 0.343 | |
| 2. Testa del Gargano |  | | interc. | -91.62 | 74.33 | -1.233 | 0.218 | | --- | --- | --- | --- | --- | | year | 0.045 | 0.037 | 1.210 | 0.226 | |  | | interc. | -268.0 | 153.4 | -1.747 | 0.081 | | --- | --- | --- | --- | --- | | year | 0.132 | 0.076 | 1.730 | 0.084 | |
| 1. Baia dei Campi |  | | interc. | 8.892 | 82.43 | 0.142 | 0.887 | | --- | --- | --- | --- | --- | | year | -0.005 | 0.031 | -0.168 | 0.867 | |  | | interc. | -198.1 | 88.08 | -2.249 | **0.025** | | --- | --- | --- | --- | --- | | year | 0.098 | 0.044 | 2.221 | **0.026** | |
| 3. Torre dei Campi |  | | interc. | 27.39 | 75.09 | 0.365 | 0.715 | | --- | --- | --- | --- | --- | | year | -0.015 | 0.037 | -0.389 | 0.697 | |  | | interc. | -209.2 | 143.6 | -1.457 | 0.145 | | --- | --- | --- | --- | --- | | year | 0.103 | 0.072 | 1.438 | 0.151 | |
| 8. Pugnochiuso |  | | interc. | 198.3 | 101.3 | 1.958 | 0.050 | | --- | --- | --- | --- | --- | | year | -0.1 | 0.051 | -1.973 | 0.050 | |  | | interc. | -3.04 | 171.4 | -0.018 | 0.986 | | --- | --- | --- | --- | --- | | year | 1-13 | 8.5e-2 | 0 | 1 | |

|  | Estim. | Std. Err. | z value | P(>|z|) |
| --- | --- | --- | --- | --- |

|  | Estim. | Std. Err. | z value | P(>|z|) |
| --- | --- | --- | --- | --- |

**Table S5.** Analysis of the dependence of the proportion of introgressed individuals of *Ae. mariae* and *Ae. zammitii* with respect to year and species by logistic regression. The sites are coded as in Fig. 1. The analyses were performed for all sites with the exception of the site 5, Vieste, where no introgression was observed in *Ae. zammitii* and the site 6, San Lorenzo, where introgression was observed only in 2011 (Table S2).

| **Site** |  | **Estim.** | **Std. Err.** | **z value** | **P(>|z|)** |
| --- | --- | --- | --- | --- | --- |
| 4. Torre del Ponte | interc. | -12.741 | 58.281 | -0.219 | 0.8269 |
|  | species | -2.648 | 0.750 | -3.533 | 0.00041*** |
|  | year | 0.006 | 0.029 | 0.193 | 0.8470 |
| 2. Testa del Gargano | interc. | -52.155 | 61.168 | -0.853 | 0.394 |
|  | species | -0.848 | 0.461 | -1.842 | 0.0455* |
|  | year | 0.025 | 0.031 | 0.824 | 0.410 |
| 1. Baia dei Campi | interc. | -98.925 | 41.936 | -2.359 | 0.0183*** |
|  | species | -1.101 | 0.412 | -2.670 | 0.0076** |
|  | year | 0.049 | 0.021 | 2.317 | 0.0205*** |
| 3. Torre del Ponte | interc. | -32.200 | 68.712 | -0.469 | 0.6394 |
|  | species | -1.124 | 0.546 | -2.057 | 0.0397* |
|  | year | 0.015 | 0.034 | 0.442 | 0.6586 |
| 8. Pugnochiuso | interc. | 166.94 | 101.802 | 1.640 | 0.1010 |
|  | species | -1.463 | 0.620 | -2.360 | 0.0183* |
|  | year | -0.0841 | 0.051 | -1.656 | 0.0977 |

**Figure S1.** Alignment of the CO I mtDNA gene fragments of *Aedes mariae* and *Ae. zammitii*. The haplotypes have been downloaded by GenBank and denoted with the letters m and z, respectively for *Aedes mariae* and *Ae. zammitii*, followed by the haplotype number. In the dark grey area the position of the restriction site of *BspLi*/*Nla*IV is shown. The accession numbers of the sequences are: m1 (KM592029); m2 (KM592030); m3 (KM592031); m4 (KM592032); m5 (KM592033); m6 (KM592034); m7 (KM592035); m8 (KM592036); m9 (KM592037); m10 (KM592038); m11 (KM592039); z1 (KM592040); z2 (KM592041); z3 (KM592042); z4 (KM592043); z5 (KM592044); z6 (KM592045); z7 (KM592046); z8 (KM592047); z9 (KM592048); z10 (KM592049); z11 (KM592050).

m9 ATTCGTGCTGAGTTAAGTCAACCTGGTATATTTATTGGAAATGACCAAATTTATAATGTA 60 bp

m11 ATTCGTGCTGAGTTAAGTCAACCTGGTATATTTATTGGAAATGACCAAATTTATAATGTA

m10 ATTCGTGCTGAATTAAGTCAACCTGGTATATTTATTGGAAATGACCAAATTTATAATGTA

m1 ATTCGTGCTGAATTAAGTCAACCTGGTATATTTATTGGAAATGACCAAATTTATAATGTA

m2 ATTCGTGCTGAATTAAGTCAACCTGGTATATTTATTGGAAATGACCAAATTTATAATGTA

m3 ATTCGTGCTGAATTAAGTCAACCTGGTATATTTATTGGAAATGACCAAATTTATAATGTA

m8 ATTCGTGCTGAATTAAGTCAACCTGGTATATTTATTGGAAATGACCAAATTTATAATGTA

m7 ATTCGTGCTGAATTAAGTCAACCTGGTATATTTATTGGAAATGACCAAATTTATAATGTA

m4 ATTCGTGCTGAATTAAGTCAACCTGGTATATTTATTGGAAATGACCAAATTTATAATGTA

m5 ATTCGTGCTGAATTAAGTCAACCTGGTATATTTATTGGAAATGACCAAATTTATAATGTA

m6 ATTCGTGCTGAATTAAGTCAACCTGGTATATTTATTGGAAATGACCAAATTTATAATGTA

z1 ATTCGTGCTGAATTAAGTCAACCAGGTATATTTATTGGAAATGACCAAATTTATAATGTA

z6 ATTCGTGCTGCATTAAGTCAACCAGGTATATTTATTGGAAATGACCAAATTTATAATGTA

z3 ATTCGTGCTGAATTAAGTCAACCAGGTATATTTATTGGAAATGACCAAATTTATAATGTA

z4 ATTCGTGCTGAATTAAGTCAACCAGGTATATTTATTGGAAATGACCAAATTTATAATGTA

z2 ATTCGTGCTGAATTAAGTCAACCAGGTATATTTATTGGAAATGACCAAATTTATAATGTA

z5 ATTCGTGCTGAATTAAGTCAACCAGGTATATTTATTGGAAATGACCAAATTTATAATGTA

z11 ATTCGTGCTGAATTAAGTCAACCAGGTATATTCATTGGAAATGACCAAATTTATAATGTA

z8 ATTCGTGCTGAATTAAGTCAACCAGGTATATTCATTGGAAATGACCAAATTTATAATGTA

z10 ATTCGTGCTGAATTAAGTCAACCAGGTATATTCATTGGAAATGACCAAATTTATAATGTA

z9 ATTCGTGCTGAATTAAGTCAACCAGGTATATTCATTGGAAATGACCAAATTTATAATGTA

z7 ATTCGTGCTGAATTAAGTCAACCAGGTATATTTATTGGAAATGACCAAATTTATAATGTA

********** *********** ******** ***************************

m9 ATTGTTACAGCTCATGCTTTTATTATAATTTTCTTTATAGTTATACCTATTATAATTGGA 120 bp

m11 ATTGTTACAGCTCATGCTTTTATTATAATTTTCTTTATAGTTATACCTATTATAATTGGA

m10 ATTGTTACAGCTCATGCTTTTATTATAATTTTCTTTATAGTTATACCTATTATAATTGGA

m1 ATTGTTACAGCTCATGCTTTTATTATAATTTTTTTTATAGTTATACCTATTATAATTGGA

m2 ATTGTTACAGCTCATGCTTTTATTATAATTTTCTTTATAGTTATACCTATTATAATTGGA

m3 ATTGTTACAGCTCATGCTTTTATTATAATTTTTTTTATAGTTATACCTATTATAATTGGA

m8 ATTGTTACAGCTCATGCTTTTATTATAATTTTTTTTATAGTTATACCTATTATAATTGGA

m7 ATTGTTACAGCTCATGCTTTTATTATAATTTTTTTTATAGTTATACCTATTATAATTGGA

m4 ATTGTTACAGCTCATGCTTTTATTATAATTTTCTTTATAGTTATACCTATTATAATTGGA

m5 ATTGTTACAGCTCATGCTTTTATTATAATTTTCTTTATAGTTATACCTATTATAATTGGA

m6 ATTGTTACAGCTCATGCTTTTATTATAATTTTCTTTATAGTTATACCTATTATAATTGGA

z1 ATTGTTACAGCTCATGCTTTTATTATAATTTTCTTTATAGTAATACCTATTATAATTGGA

z6 ATTGTTACAGCTCATGCTTTTATTATAATTTTCTTTATAGTAATACCTATTATAATTGGA

z3 ATTGTTACAGCTCATGCTTTTATTATAATTTTCTTTATAGTAATACCTATTATAATTGGA

z4 ATTGTTACAGCTCATGCTTTTATTATAATTTTCTTTATAGTAATACCTATTATAATTGGA

z2 ATTGTTACAGCTCATGCTTTTATTATAATTTTCTTTATAGTAATACCTATTATAATTGGA

z5 ATTGTTACAGCTCATGCTTTTATTATAATTTTCTTTATAGTAATACCTATTATAATTGGA

z11 ATTGTTACAGCTCATGCTTTTATTATAATTTTCTTTATAGTAATACCTATTATAATTGGA

z8 ATTGTTACAGCTCATGCTTTTATTATAATTTTCTTTATAGTAATACCTATTATAATTGGA

z10 ATTGTTACAGCTCATGCTTTTATTATAATTTTCTTTATAGTAATACCTATTATAATTGGA

z9 ATTGTTACAGCTCATGCTTTTATTATAATTTTCTTTATAGTAATACCTATTATAATTGGA

z7 ATTGTTACAGCTCATGCTTTTATTATAATTTTCTTTATAGTAATACCTATTATAATTGGA

******************************** ******** ******************

m9 GGATTTGGAAATTGATTAGTTCCTTTAATATTAGGAGCACCAGATATGGCATTTCCTCGA 180 bp

m11 GGATTTGGAAATTGATTAGTTCCTTTAATATTAGGAGCACCAGATATGGCATTTCCTCGA

m10 GGATTTGGAAATTGATTAGTTCCTTTAATATTAGGAGCACCAGATATGGCATTTCCTCGA

m1 GGATTTGGAAATTGATTAGTTCCTTTAATATTAGGAGCACCAGATATAGCATTTCCTCGA

m2 GGATTTGGAAATTGATTAGTTCCTTTAATATTAGGAGCACCAGATATAGCATTTCCTCGA

m3 GGATTTGGAAATTGATTAGTTCCTTTAATATTAGGAGCACCAGATATAGCATTTCCTCGA

m8 GGATTTGGAAATTGATTAGTTCCTTTAATATTAGGAGCACCAGATATAGCATTTCCTCGA

m7 GGATTTGGAAATTGATTAGTTCCTTTAATATTAGGAGCACCAGATATAGCATTTCCTCGA

m4 GGATTTGGAAATTGATTAGTTCCTTTAATATTAGGAGCACCAGATATAGCATTTCCTCGA

m5 GGATTTGGAAATTGATTAGTTCCTTTAATATTAGGAGCACCAGATATAGCATTTCCTCGA

m6 GGATTTGGAAATTGATTAGTTCCTTTAATATTAGGAGCACCAGATATAGCATTTCCTCGA

z1 GGATTTGGAAATTGATTAGTTCCTTTAATATTAGGAGCACCAGACATAGCGTTTCCTCGA

z6 GGATTTGGAAATTGATTAGTTCCTTTAATATTAGGAGCACCAGACATAGCGTTTCCTCGA

z3 GGATTTGGAAATTGATTAGTTCCTTTAATATTAGGAGCACCAGACATAGCGTTTCCTCGA

z4 GGATTTGGAAATTGATTAGTTCCTTTAATATTAGGAGCACCAGACATAGCATTTCCTCGA

z2 GGATTTGGAAATTGATTAGTTCCTTTAATATTAGGAGCACCAGACATAGCATTTCCTCGA

z5 GGATTTGGAAATTGATTAGTTCCTTTAATATTAGGAGCACCAGACATAGCATTTCCTCGA

z11 GGATTTGGAAATTGATTAGTTCCTTTAATATTAGGAGCACCAGATATAGCATTTCCTCGA

z8 GGATTTGGAAATTGATTAGTTCCTTTAATATTAGGAGCACCAGACATAGCATTTCCTCGA

z10 GGATTTGGAAATTGATTAGTTCCTTTAATATTAGGAGCACCAGACATAGCATTTCCTCGA

z9 GGATTTGGAAATTGATTAGTTCCTTTAATATTAGGAGCACCAGACATAGCATTTCCTCGA

z7 GGATTTGGAAATTGATTAGTTCCTTTAATATTAGGAGCACCAGACATAGCATTTCCTCGA

******************************************** ** ** *********

m9 ATAAATAATATAAGTTTTTGAATATTACCTCCTTCATTAACACTACTACTTTCAAGTAGT 240 bp

m11 ATAAATAATATAAGTTTTTGAATATTACCTCCTTCATTAACACTACTACTTTCAAGTAGT

m10 ATAAATAATATAAGTTTTTGAATATTACCTCCTTCATTAACACTACTACTTTCAAGTAGT

m1 ATAAATAATATAAGTTTTTGAATATTACCTCCTTCACTGACACTACTACTTTCAAGTAGT

m2 ATAAATAATATAAGTTTTTGAATATTACCTCCTTCACTGACACTACTACTTTCAAGTAGT

m3 ATAAATAATATAAGTTTTTGAATATTACCCCCTTCATTAACACTACTACTTTCAAGTAGT

m8 ATAAATAATATAAGTTTTTGAATATTACCCCCTTCATTGACACTACTACTTTCAAGTAGT

m7 ATAAATAATATAAGTTTTTGAATATTACCTCCTTCATTGACACTACTACTTTCAAGTAGT

m4 ATAAATAATATAAGTTTTTGAATATTACCTCCTTCATTGACACTACTACTTTCAAGTAGT

m5 ATAAATAATATAAGTTTTTGAATATTACCCCCTTCATTGACACTACTACTTTCAAGTAGT

m6 ATAAATAATATAAGTTTTTGAATATTACCTCCTTCATTGACACTACTACTTTCAAGTAGT

z1 ATAAATAATATAAGTTTTTGAATACTACCTCCTTCATTAACACTGCTACTCTCAAGTAGT

z6 ATAAATAATATAAGTTTTTGAATACTACCTCCTTCATTAACACTGCTACTCTCAAGTAGT

z3 ATAAATAATATAAGTTTTTGAATACTACCTCCTTCATTAACACTGCTACTCTCAAGTAGT

z4 ATAAATAATATAAGTTTTTGAATACTACCTCCTTCATTAACACTGCTACTCTCAAGTAGT

z2 ATAAATAATATAAGTTTTTGAATACTACCCCCTTCATTGACACTGCTACTCTCAAGTAGT

z5 ATAAATAATATAAGTTTTTGAATACTACCTCCTTCATTGACACTGCTACTCTCAAGTAGT

z11 ATAAACAATATAAGTTTTTGAATACTGCCTCCTTCATTAACACTCCTACTCTCAAGTAGT

z8 ATAAATAATATAAGTTTTTGAATATTACCTCCTTCATTAACACTACTACTATCAAGTAGT

z10 ATAAATAATATAAGTTTTTGAATATTACCTCCTTCATTAACACTGCTACTATCAAGTAGT

z9 ATAAATAATATAAGTTTTTGAATATTACCTCCTTCATTAACACTGCTACTCTCAAGTAGT

z7 ATAAATAATATAAGTTTTTGAATATTACCTCCTTCATTAACACTGCTACTCTCAAGTAGT

***** ****************** * ** ****** * ***** ***** *********

m9 ATAGTAGAAAATGGATCAGGAACTGGATGAACAGTTTATCCACCTCTTTCATCTGGAACT 300 bp

m11 ATAGTAGAAAATGGATCAGGAACTGGATGAACAGTTTATCCACCTCTTTCATCTGGAACT

m10 ATAGTAGAAAATGGATCAGGAACTGGATGAACAGTTTATCCACCTCTTTCATCTGGAACT

m1 ATAGTAGAAAATGGATCAGGAACTGGATGAACAGTTTATCCACCTCTTTCATCTGGAACT

m2 ATAGTAGAAAATGGATCAGGAACTGGATGAACAGTTTATCCACCTCTTTCATCTGGAACT

m3 ATAGTAGAAAATGGATCAGGAACTGGATGAACAGTTTATCCACCTCTTTCATCTGGAACT

m8 ATAGTAGAAAATGGATCAGGAACTGGATGAACAGTTTATCCACCTCTTTCATCTGGAACT

m7 ATAGTAGAAAATGGATCAGGAACTGGATGAACAGTTTATCCACCTCTTTCATCTGGAACT

m4 ATAGTAGAAAATGGATCAGGAACTGGATGAACAGTTTATCCACCTCTTTCATCTGGAACT

m5 ATAGTAGAAAATGGATCAGGAACTGGATGAACAGTTTATCCACCTCTTTCATCTGGAACT

m6 ATAGTAGAAAATGGATCAGGAACTGGATGAACAGTTTATCCACCTCTTTCATCTGGAACT

z1 ATAGTAGAAAATGGATCAGGAACAGGGTGAACAGTTTATCCTCCTCTTTCATCTGGAACT

z6 ATAGTAGAAAATGGATCAGGAACAGGGTGAACAGTTTATCCTCCTCTTTCATCTGGAACT

z3 ATAGTAGAAAATGGATCAGGAACAGGATGAACAGTTTATCCCCCTCTTTCATCTGGAACT

z4 ATAGTAGAAAATGGATCAGGAACAGGATGAACAGTTTATCCCCCTCTTTCATCTGGAACT

z2 ATAGTAGAAAATGGATCAGGAACAGGATGAACAGTTTATCCCCCTCTTTCATCTGGAACT

z5 ATAGTAGAAAATGGATCAGGAACAGGATGAACAGTTTATCCCCCTCTTTCATCTGGAACT

z11 ATAGTAGAAAATGGATCAGGAACAGGATGAACAGTTTATCCCCCTCTTTCGTCTGGAACT

z8 ATAGTAGAAAATGGATCAGGAACAGGGTGAACAGTTTACCCTCCTCTTTCATCTGGAACT

z10 ATAGTAGAAAATGGATCAGGAACAGGGTGAACAGTTTACCCTCCTCTTTCATCTGGAACT

z9 ATAGTAGAAAATGGATCAGGAACAGGGTGAACAGTTTATCCTCCTCTTTCATCTGGAACT

z7 ATAGTAGAAAATGGATCAGGAACAGGGTGAACAGTTTATCCTCCTCTTTCATCTGGAACT

*********************** ** *********** ** ******** *********

m9 GCCCATGCA**GGAGCC**TCTGTTGATTTAACAATTTTTTCTTTACATTTAGCAGGAGTATCA 360 bp

m11 GCCCATGCA**GGAGCC**TCTGTTGATTTAACAATTTTTTCTTTACATTTAGCAGGAGTATCA

m10 GCCCATGCA**GGAGCC**TCTGTTGATTTAACAATTTTTTCTTTACATTTAGCAGGAGTATCA

m1 GCCCATGCA**GGAGCC**TCTGTTGATTTAACAATTTTTTCTTTACATTTAGCAGGAGTATCA

m2 GCCCATGCA**GGAGCC**TCTGTTGATTTAACAATTTTTTCTTTACATTTAGCAGGAGTATCA

m3 GCCCATGCA**GGAGCC**TCTGTTGATTTAACAATTTTTTCTTTACATTTAGCAGGAGTATCA

m8 GCCCATGCA**GGAGCC**TCTGTTGATTTAACAATTTTTTCTTTACATTTAGCAGGAGTATCA

m7 GCCCATGCA**GGAGCC**TCTGTTGATTTAACAATTTTTTCTTTACATTTAGCAGGAGTATCA

m4 GCCCATGCA**GGAGCC**TCTGTTGATTTAACAATTTTTTCTTTACATTTAGCAGGAGTATCA

m5 GCCCATGCA**GGAGCC**TCTGTTGATTTAACAATTTTTTCTTTACATTTAGCAGGAGTATCA

m6 GCCCATGCA**GGAGCC**TCTGTTGATTTAACAATTTTTTCTTTACATTTAGCAGGAGTATCA

z1 GCTCATGCA**GGAGCT**TCAGTTGATTTAACAATTTTTTCTTTACATTTAGCAGGAGTATCA

z6 GCTCATGCA**GGAGCT**TCAGTTGATTTAACAATTTTTTCTTTACATTTAGCAGGAGTATCA

z3 GCTCATGCA**GGAGCT**TCAGTTGATTTAACAATTTTTTCTTTACATTTAGCAGGAGTATCA

z4 GCTCATGCA**GGAGCT**TCAGTTGATTTAACAATTTTTTCTTTACATTTAGCAGGAGTATCA

z2 GCTCATGCA**GGAGCT**TCAGTTGATTTAACAATTTTTTCTTTACATTTAGCAGGAGTATCA

z5 GCTCATGCA**GGAGCT**TCAGTTGATTTAACAATTTTTTCTTTACATTTAGCAGGAGTATCA

z11 GCTCATGCA**GGAGCT**TCAGTTGATTTAACAATTTTTTCTTTACATTTAGCAGGAGTATCA

z8 GCTCATGCA**GGAGCT**TCAGTTGATTTAACAATTTTTTCTTTACATTTAGCAGGAGTATCC

z10 GCTCATGCA**GGAGCT**TCAGTTGATTTAACAATTTTTTCTTTACATTTAGCAGGAGTATCA

z9 GCTCATGCA**GGAGCT**TCAGTTGATTTAACAATTTTTTCTTTACATTTAGCAGGAGTATCA

z7 GCTCATGCA**GGAGCT**TCAGTTGATTTAACAATTTTTTCTTTACATTTAGCAGGAGTATCA

** *********** ** *****************************************

m9 TCAATTTTAGGAGCAGTAAATTTTATTACTACTGTTATTAATATACGATCAGCAGGAATT 420 bp

m11 TCAATTTTAGGAGCAGTAAATTTTATTACTACTGTTATTAATATACGATCAGCAGGAATT

m10 TCAATTTTAGGAGCAGTAAATTTTATTACTACTGTTATTAATATACGATCAGCAGGAATT

m1 TCAATTTTAGGAGCAGTAAATTTTATTACTACTGTTATTAATATACGATCAGCAGGAATT

m2 TCAATTTTAGGAGCAGTAAATTTTATTACTACTGTTATTAATATACGATCAGCAGGAATT

m3 TCAATTTTAGGAGCAGTAAATTTTATTACTACTGTTATTAATATACGATCAGCAGGAATT

m8 TCAATTTTAGGAGCAGTAAATTTTATTACTACTGTTATTAATATACGATCAGCAGGAATT

m7 TCAATTTTAGGAGCAGTAAATTTTATTACTACTGTTATTAATATACGATCAGCAGGAATT

m4 TCAATTTTAGGAGCAGTAAATTTTATTACTACTGTTATTAATATACGATCAGCAGGAATT

m5 TCAATTTTAGGAGCAGTAAATTTTATTACTACTGTTATTAATATACGATCAGCAGGAATT

m6 TCAATTTTAGGAGCAGTAAATTTTATTACTACTGTTATTAATATACGATCAGCAGGAATT

z1 TCAATTTTAGGAGCAGTAAATTTTATTACTACTGTTATTAATATACGATCAGCAGGAATT

z6 TCAATTTTAGGAGCAGTAAATTTTATTACTACTGTTATTAATATACGATCAGCAGGAATT

z3 TCAATTTTAGGAGCAGTAAATTTTATTACTACTGTTATTAATATACGATCAGCAGGAATT

z4 TCAATTTTAGGAGCAGTAAATTTTATTACTACTGTTATTAATATACGATCAGCAGGAATT

z2 TCAATTTTAGGAGCAGTAAATTTTATTACTACTGTTATTAATATACGATCAGCAGGAATT

z5 TCAATTTTAGGAGCAGTAAATTTTATTACTACTGTTATTAATATACGATCAGCAGGAATT

z11 TCAATTTTAGGAGCAGTAAATTTTATTACTACTGTTATTAATATACGATCAGCAGGAATT

z8 TCAATTTTAGGAGCAGTAAATTTTATTACTACTGTTATTAATATACGATCAGCAGGAATT

z10 TCAATTTTAGGAGCAGTAAATTTTATTACTACTGTTATTAATATACGATCAGCAGGAATT

z9 TCAATTTTAGGAGCAGTAAATTTTATTACTACTGTTATTAATATACGATCAGCAGGAATT

z7 TCAATTTTAGGAGCAGTAAATTTTATTACTACTGTTATTAATATACGATCAGCAGGAATT

************************************************************

m9 ACATTAGATCGATTACCTTTATTTGTTTGATCTGTTGTAATTACAGCTGTATTATTACTT 480 bp

m11 ACATTAGATCGATTACCTTTATTTGTTTGATCTGTTGTAATTACAGCTGTATTATTACTT

m10 ACATTAGATCGATTACCTTTATTTGTTTGATCTGTTGTAATTACAGCTGTATTATTACTT

m1 ACATTAGATCGATTACCTTTATTTGTTTGATCTGTTGTAATTACAGCTGTATTATTACTT

m2 ACATTAGATCGATTACCTTTATTTGTTTGATCTGTTGTAATTACAGCTGTATTATTACTT

m3 ACATTAGATCGATTACCTTTATTTGTTTGATCTGTTGTAATTACAGCTGTATTATTACTT

m8 ACATTAGATCGATTACCTTTATTTGTTTGATCTGTTGTAATTACAGCTGTATTATTACTT

m7 ACATTAGATCGATTACCTTTATTTGTTTGATCTGTTGTAATTACAGCTGTATTATTACTT

m4 ACATTAGATCGATTACCTTTATTTGTTTGATCTGTTGTAATTACAGCTGTATTATTACTT

m5 ACATTAGATCGATTACCTTTATTTGTTTGATCTGTTGTAATTACAGCTGTATTATTACTT

m6 ACATTAGATCGATTACCTTTATTTGTTTGATCTGTTGTAATTACAGCTGTATTATTACTT

z1 ACATTAGATCGATTACCTTTATTTGTTTGATCTGTTGTAATTACAGCTGTATTATTACTT

z6 ACATTAGATCGATTACCTTTATTTGTTTGATCTGTTGTAATTACAGCTGTATTATTACTT

z3 ACATTAGATCGATTACCTTTATTTGTTTGATCTGTTGTAATTACAGCTGTATTATTACTT

z4 ACATTAGATCGATTACCTTTATTTGTTTGATCTGTTGTAATTACAGCTGTATTATTACTT

z2 ACATTAGATCGATTACCTTTATTTGTTTGATCTGTTGTAATTACAGCTGTATTATTACTT

z5 ACATTAGATCGATTACCTTTATTTGTTTGATCTGTTGTAATTACAGCTGTATTATTACTT

z11 ACATTAGATCGATTACCTTTATTTGTTTGATCTGTTGTAATTACAGCTGTATTATTACTT

z8 ACATTAGATCGATTACCTTTATTTGTTTGATCTGTTGTAATTACAGCTGTATTATTACTT

z10 ACATTAGATCGATTACCTTTATTTGTTTGATCTGTTGTAATTACAGCTGTATTATTACTT

z9 ACATTAGATCGATTACCTTTATTTGTTTGATCTGTTGTAATTACAGCTGTATTATTACTT

z7 ACATTAGATCGATTACCTTTATTTGTTTGATCTGTTGTAATTACAGCTGTATTATTACTT

************************************************************

m9 TTATCATTACCTGTTTTAGCTGGAGCTATTACTATATTATTAACTGATCGAAATTTAAAT 540 bp

m11 TTATCATTACCTGTTTTAGCTGGAGCTATTACTATATTATTAACTGATCGAAATTTAAAT

m10 TTATCATTACCTGTTTTAGCTGGAGCTATTACTATATTATTAACTGATCGAAATTTAAAT

m1 TTATCATTACCTGTTTTAGCTGGAGCTATTACTATATTATTAACTGATCGAAATTTAAAT

m2 TTATCATTACCTGTTTTAGCTGGAGCTATTACTATATTATTAACTGATCGAAATTTAAAT

m3 TTATCATTACCTGTTTTAGCTGGAGCTATTACTATATTATTAACTGATCGAAATTTAAAT

m8 TTATCATTACCTGTTTTAGCTGGAGCTATTACTATATTATTAACTGATCGAAATTTAAAT

m7 TTATCATTACCTGTTTTAGCTGGAGCTATTACTATATTATTAACTGATCGAAATTTAAAT

m4 TTATCATTACCTGTTTTAGCTGGAGCTATTACTATATTATTAACTGATCGAAATTTAAAT

m5 TTATCATTACCTGTTTTAGCTGGAGCTATTACTATATTATTAACTGATCGAAATTTAAAT

m6 TTATCATTACCTGTTTTAGCTGGAGCTATTACTATATTATTAACTGATCGAAATTTAAAT

z1 TTATCATTACCTGTTTTAGCTGGAGCTATTACTATATTATTAACTGATCGAAATTTAAAT

z6 TTATCATTACCTGTTTTAGCTGGAGCTATTACTATATTATTAACTGATCGAAATTTAAAT

z3 TTATCATTACCTGTTTTAGCTGGAGCTATTACTATATTATTAACTGATCGAAATTTAAAT

z4 TTATCATTACCTGTTTTAGCTGGAGCTATTACTATATTATTAACTGATCGAAATTTAAAT

z2 TTATCATTACCTGTTTTAGCTGGAGCTATTACTATATTATTAACTGATCGAAATTTAAAT

z5 TTATCATTACCTGTTTTAGCTGGAGCTATTACTATATTATTAACTGATCGAAATTTAAAT

z11 TTATCATTACCTGTTTTAGCTGGAGCTATTACTATATTATTAACTGATCGAAATTTAAAT

z8 TTATCATTACCTGTTTTAGCTGGAGCTATTACTATATTATTAACAGATCGAAATTTAAAT

z10 TTATCATTACCTGTTTTAGCTGGAGCTATTACTATATTATTAACAGATCGAAATTTAAAT

z9 TTATCATTACCTGTTTTAGCTGGAGCTATTACTATATTATTAACAGATCGAAATTTAAAT

z7 TTATCATTACCTGTTTTAGCTGGAGCTATTACTATATTATTAACAGATCGAAATTTAAAT

******************************************** ***************

m9 ACTTCATTCTTTGATCCTATTGGAGGAGGAGATCCTATTTTATACCAACATTTA 594 bp

m11 ACTTCATTCTTTGATCCTATTGGAGGAGGAGACCCTATTTTATACCAACATTTA

m10 ACTTCATTCTTTGATCCTATTGGAGGAGGAGATCCTATTTTATACCAACATTTA

m1 ACTTCATTCTTTGATCCTATTGGAGGAGGAGATCCTATTTTATATCAACATTTA

m2 ACTTCATTCTTTGATCCTATTGGAGGAGGAGATCCTATTTTATATCAACATTTA

m3 ACTTCATTCTTTGATCCTATTGGAGGAGGAGATCCTATTTTATACCAACATTTA

m8 ACTTCATTCTTTGATCCTATTGGAGGAGGAGATCCTATTTTATACCAACATTTA

m7 ACTTCATTCTTTGATCCTATTGGAGGAGGAGATCCTATTTTATACCAACATTTA

m4 ACTTCATTCTTTGACCCTATTGGAGGAGGAGATCCTATTTTATACCAACATTTA

m5 ACTTCATTCTTTGACCCTATTGGAGGAGGAGATCCTATTTTATACCAACATTTA

m6 ACTTCATTCTTTGATCCTATTGGAGGAGGAGATCCTATTTTATACCAACATTTA

z1 ACTTCATTTTTTGATCCTATTGGAGGAGGAGACCCTATTTTATATCAACATTTA

z6 ACTTCATTTTTTGATCCTATTGGAGGAGGAGACCCTATTTTATATCAACATTTA

z3 ACTTCATTTTTTGATCCTATTGGAGGAGGAGACCCTATTTTATATCAACATTTA

z4 ACTTCATTTTTTGATCCTATTGGAGGAGGAGACCCTATTTTATACCAACATTTA

z2 ACTTCATTTTTTGATCCTATTGGAGGAGGAGACCCTATTTTATACCAACATTTA

z5 ACTTCATTTTTTGATCCTATTGGAGGAGGAGACCCTATTTTATACCAACATTTA

z11 ACTTCATTTTTTGATCCTATTGGAGGAGGAGACCCTATTTTATACCAACATTTA

z8 ACTTCATTTTTTGATCCTATTGGAGGAGGAGATCCTATTTTATACCAACATTTA

z10 ACTTCATTTTTTGATCCTATTGGAGGAGGAGATCCTATTTTATACCAACATTTA

z9 ACTTCATTTTTTGATCCTATTGGAGGAGGAGATCCTATTTTATACCAACATTTA

z7 ACTTCATTTTTTGATCCTATTGGAGGAGGAGATCCTATTTTATATCAACATTTA

******** ***** ***************** *********** *********
